# Psychological Well-Being and Self-Aging Attitudes Moderate the Association between Subjective Age and Age Discrimination in the Workplace

**DOI:** 10.3390/bs14090742

**Published:** 2024-08-25

**Authors:** Assaf Suberry, Ehud Bodner

**Affiliations:** 1 Department of Social & Health Sciences, Bar-Ilan University, Ramat Gan 5290002, Israel; ehud.bodner@biu.ac.il; 2 Music Department, Bar-Ilan University, Ramat Gan 5290002, Israel

**Keywords:** age discrimination in the workplace, ageism, positive psychology, psychological well-being, subjective age

## Abstract

Views of aging include peoples’ assessment of their own aging process and their subjective age. Positive views of aging relate to a improved psychological well-being which predicts better physical and mental health. While these relationships were substantially studied, the moderating roles of self-aging attitudes and psychological well-being in the subjective age–age discrimination connection have been much less explored. The current study used a convenience sample of 568 participants (mean = 66.21y, SD = 11.95, age range 50–95), 55.8% women, 67.1% employed. In line with the hypotheses, young subjective age and psychological well-being were connected to less age discrimination in the workplace, and higher psychological well-being mitigated the subjective age–age discrimination at work connection. When the perception of old age as a period of loss was added to the model, adults who perceived old age as a period of loss and reported lower levels of psychological well-being demonstrated the strongest relationship between an increase in subjective age and an increase in age-related discrimination at work. The findings emphasize the importance of the psychological well-being of older employees as a resource for improving their attitudes towards their last years at work.

## 1. Introduction

The world’s older population is steadily increasing, and the proportion of those aged 60 and over is expected to almost double from 12% to 22% by the year 2050 [1]. In light of these demographic changes, and in response to the increase in the number of older employees and the lengthening of their working years, researchers have begun to take an interest in the associations linking employee’s age, work productivity, and psychological well-being with people’s attitudes towards older workers (for a review, see [2]).

Ageism, the way people think (stereotypes), feel (prejudices), and behave (discrimination) towards others because of their older age, is a common disturbing social phenomenon [3]. An older person may often be perceived as incompetent, slow, and fragile [4]. According to the Stereotype Embodiment theory [5], ageist attitudes are prevalent in society and are internalized from childhood, due to continuous exposure to common beliefs and prejudice held towards older adults. These perceptions might even be directed towards oneself when a person reaches old age. This phenomenon of ‘self-ageism’ is linked to the way the individual perceives aging in general, and to his or her personal aging experience. Such negative aging perceptions may adversely affect the physical health and well-being of older adults, as well as their cognitive functions and longevity [4,6].

**Age-related discrimination in the workplace.** Ageism in the workplace is manifested in individual or institutional (formal or informal) labeling and discrimination against employees due to their advanced age. Despite the fact that discrimination which is based on age is prohibited by law in many countries (for example in Israel; [7]), and in spite of the difficulty of estimating the exact extent of this type of discrimination, it seems to be a widespread phenomenon. The percentage of employees who experience discrimination at work ranges from 48.1% [8] to 91% [9]. MIDUS (Midlife in the US), an extensive longitudinal ongoing survey on old age conducted in the US, indicated that 81% of employed people aged 50 and over reported experiencing age discrimination in the past year [10]. Recently, age discrimination in the high-tech industry has also been reported. An accelerated momentum of automation and digitization at the workplace, caused by the COVID-19 pandemic has resulted in an increase in the threshold conditions for positions in industry. This, in turn, has made it harder for the middle-aged population to enter the labor market [11]. In addition, hiring managers who tend to rate programmers aged 45 and older as less suitable for the job, may also stem from the assumption that older workers are unable or are unwilling to try new technologies [12].

In general, older employees are perceived as being inflexible, and as tending to avoid changes, or as having low ability to acquire new skills compared with younger workers [13]. It was also found that managers with negative ageist views tend to think that older workers should retire earlier [14]. Age discrimination manifests itself in the prevention of promotion in the workplace and a general preference for hiring young people [15,16]. On the other hand, it was found that older workers are positively labeled as more loyal and trustworthy compared to their younger colleagues [17]. These findings are worrisome considering the increasing proportion of older workers, which will expose a cumulative ratio of older employees to negative and discriminatory treatment.

The current study focuses on a specific aspect of ageism at work: older employees’ subjective attitudes and perceptions of age discrimination in the workplace. Studies have found negative relationships between perceived age discrimination and self-efficacy [18], organizational commitment, work involvement, and life and work satisfaction [19,20]. In addition, it was found that the more the employees felt discriminated against at work due to their age, the more they reported a tendency to look for other ways of earning a living or to retire from work [21]. In general, discrimination at work—of any kind—is a stressful factor, which is associated with increased levels of depression and anxiety, as well as with reduced levels of psychological well-being and life satisfaction [22,23].

**Figure 1:**
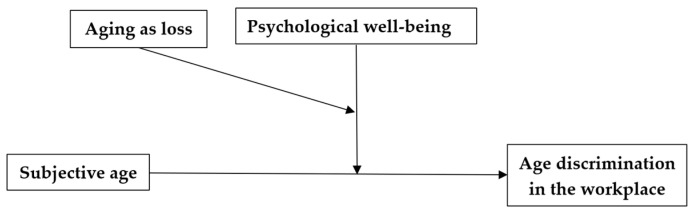
The Research Model for Hypothesis 6 Describing Two Moderation Effects.

Do employees with lower levels of psychological well-being perceive themselves as being more discriminated against at work because of age? Can negative and positive perceptions of aging lead to higher and lower (respectively) levels of self-perceptions of age discrimination in the workplace? Can the combined effect of psychological resources, such as positive perceptions of aging and psychological well-being, affect self-perceptions of age discrimination at work? These questions will be explored later, and the study will analyze a conceptual model incorporating six research hypotheses, along with a moderated moderation model [24] (see Figure 1; at the end of the Introduction section).

**Psychological well-being and age discrimination in the workplace.** Psychological well-being is a broad term reflecting the existential challenges that individuals face and their potential for optimal functioning in life. The World Health Organization highlights the importance of psychological well-being. The organization thus conceptualized it as a foundational resource that empowers individuals to reach their full personal and social potential. It also helps them manage life and work challenges [25]. Carol Ryff described this concept using a model consisting of six dimensions: autonomy, personal growth and development, environmental mastery, positive relationships with others, purpose and meaning in life, and self-acceptance [26,27].

As chronological age increases, research shows that personal growth, as well as purpose and meaning in life measures decrease [26,28,29]. Despite the decline in psychological well-being in the second half of life, positive functioning with regard to meaning and purpose over the life course, together with the capacity for self-acceptance, both have a positive effect on employed people, reducing the chance of illness and improving quality of life [30,31,32].

Psychological well-being has been extensively studied globally in recent decades. The well-established body of research examined the link between psychological well-being and physical health (e.g., [33,34]), with comparatively less attention directed towards investigating the link between psychological well-being and ageism. Effective intervention programs were developed in the spirit of the Ryff’s model [35,36], and some were effectively implemented in workplaces [37]. Longitudinal studies revealed that a positive relationship exists between psychological well-being and mental and physical health [38,39]. One longitudinal study on 5500 Americans (65.7% still working) found that among 51–56-year-olds, who reported lower levels of psychological well-being, there is increased probability to develop depression after a decade [40]. According to a study conducted specifically among older women with high psychological well-being which was connected with positive biological indicators such as low cortisol values (a proxy measure of stress), low risk of heart disease, and enhanced sleep quality were found [41]. Similarly, a review study that sampled over 136,000 participants, whose average age was 67, found that among those who scored high on the purpose-in-life component, there were lower rates of heart disease and mortality [42]. Taking these studies into account, it would seem that psychological well-being contributes to a better quality of life, and perhaps even to an extended life expectancy. In addition, higher employees’ psychological well-being has also been associated with effective functioning and higher productivity of organizations and workplaces [43,44,45]. Therefore, organizations and institutions have begun to work towards the advancement of employees’ psychological well-being through changes in public policy [46].

Research has found negative effects of discrimination on psychological well-being. Not limited to age discrimination, in the broader context of prejudice (e.g., racism, sexism, and attitudes towards other minority groups), people who experience discrimination suffer from decreased self-esteem, increased psychological distress, and decreased life satisfaction, as well as from increased depression and anxiety (see [47,48]) for meta-analysis]. Conversely, in the context of ageism, discrimination at work was associated with job insecurity which, in turn, was found to be associated with lower psychological well-being [49], and positive work perceptions predicted higher psychological well-being [50].

Lately, Sanders et al. [51] criticized the limited evidence in the literature for the ability of the eudaemonist perspective to predict various sorts of prejudice, which has been left largely untested and somewhat contradictory. Indeed, the scanty and yet growing literature on the role of positive psychology concepts in predicting various sorts of prejudice (i.e., gender and race) is contradictory. For example, in contrast to the hypothesis that happiness leads to less interest in social change, it was found that positive mood predicts greater social action in several political contexts [52]. Additionally, awe (i.e., sense of wonder and respect inspired by a vast and powerful experience) was found to be related to prosocial behavior [53]. However, in contrast to these findings, meaning in life was found to be related positively to right-wing authoritarianism [54], which is a predictor of prejudice, e.g., [55,56,57]. 

In line with these developments in the literature, Sanders et al. [51] recommended building a case for the potential role of various positive psychology concepts as predictors of prejudice, to better understand these contradictions. They argued for the theoretical premise the eudaemonist perspective suggests (e.g., meaning in life, life satisfaction, happiness, awe) that it may negate prejudice and discrimination, and that therefore, various positive psychology concepts might serve as negative predictors for prejudice and discrimination. Following this last development in the literature on prejudice, we focused in this study on the umbrella term of “psychological wellbeing”, which is based on the eudaemonist perspective. We tried to better understand if moral goodness and eudaimonic well-being may serve protective roles in moderating the negative effect that an older subjective age and viewing aging as a loss might have on self-reported discrimination in the workplace. This kind of discrimination is related to a social domain which can be more prone to age discrimination as people age [58].

In conclusion, the existing research has shown that lower levels of discrimination at work are negatively associated with stress, with insecurity, and with the tendency to retire from work; and are positively associated with appreciation of older employees, with commitment to work, and with increased life satisfaction. Therefore, our first research hypothesis is that in the second half of life, higher levels of psychological well-being would be associated with less reported age discrimination at work.

**Subjective age and age discrimination in the workplace.** Young subjective age (i.e., self-assessment of one’s age as younger than one’s chronological age) is a resource used by older adults to help them cope with challenges, such as health problems, losses, and negative cultural views on older adults [59,60]. An extensive review of studies carried out in 148 countries shows that, in general, people aged 40 and over attribute themselves a younger age [61]. The reasons for this attribution stem from biological (physiological condition) and socio-psychological motives (the way society and the individual perceive old age and self-aging, and the degree of exposure to age discrimination, which is a social stress factor) [62]. From the biological perspective, a younger subjective age is considered a marker of healthy aging [62]. For instance, visual disfluency has been shown to increase subjective age [63], and stressful problems were correlated with an older subjective age, as evidenced by longitudinal and daily diary studies [64,65,66]. From a socio-psychological perspective, individuals rely on social cognitive age representations as a benchmark for determining whether their aging trajectory aligns with perceptions of youth or old age [67], and as they age, they maintain their self-esteem by disassociating from their age groups. Therefore, they often align their self-perceptions with prevailing cultural notions of aging, which are shaping their sense of being younger or older [59].

Young subjective age has been associated with better subjective and objective physiological and cognitive performance, and with better health, longevity, and psychological well-being [68]. Likewise, a younger subjective age is also associated with higher levels of life satisfaction [69] and predicts higher levels of psychological well-being [70].

In recent years, there has been an interest in the empirical and theoretical research about employees’ perceptions of their subjective age. Research shows that young subjective age has a positive effect on motivation at work and contributes to easing stress—both at work and outside of work [67,71]. A longitudinal study found that a decrease in subjective age predicted an increase in control and motivation at work [72]. Moreover, a daily diary study reported that older employees, who attributed an older subjective age to themselves, tended to attribute negative work events to their chronological age (“It happened because of my age”). In this daily diary study, workers with a younger subjective age attributed fewer negative daily events to their age and reported higher levels of attachment and commitment to work, together with increased psychological well-being [73]. Therefore, our second research hypothesis is that a younger subjective age would be associated with less reported perceived age discrimination at work.

**Aging as loss and age discrimination in the workplace.** Various theories have dealt with the psychosocial process of aging in view of the physical and social losses that adults must cope with (e.g., [74,75,76]). The Socioemotional Selectivity Theory [75] proposes that as individuals age, their temporal perspective is restricted and shifts from future-oriented to present-focused, and that this loss of time horizons motivates them to cope by selecting fewer meaningful friends and fewer meaningful activities and develop a richer sense of emotional complexity, by combining positive and negative emotional experiences. The Strength and Vulnerability (SAVI) model [76] suggests that older adults cope with the prolongation of their physical and emotional recovery time by avoiding unnecessary interpersonal conflicts. Following these two theories, we suggest that older employees may use such coping mechanisms when dealing with age discrimination. They may employ avoidance strategies to minimize exposure to negative conflicts [77], thereby reducing the emotional strain associated with discriminatory situations and better regulate their emotional reactions [78]. Nevertheless, when the conflicts in their workplace are difficult for them to avoid, they may experience difficulties in recovering their neurological hypersensitivity, which can hinder their ability to effectively regulate emotions and suffer long-term reactions [79].

The main insights that can be derived from these two theories are that the shortening of time remained to live, and the constant exposure to the natural losses that often accompany the aging process (e.g., death of loved ones, cognitive and physical decline), all lead to a complex coping and adaptation process. Since in this work we were trying to predict the negative subjective experience of age-based workplace discrimination, we focus on the loss dimension in capturing the psychological distress associated with the subjective perception of age workplace discrimination. We suggest that the view of one’s own aging as a period of losses may affect the way people experience age discrimination at work. We assume that when older employees unsuccessfully attempt to avoid events that will expose them to negative stimuli (such as age-related discrimination regarding their work performance), they may experience a great deal of vulnerability. When they fail to avoid such stimuli, they may self-internalize the negative labeling associated with “older employees”. Indeed, ageism can be perceived as unfair and obstructive to career advancement, triggering negative emotional responses including anger, anxiety, depression, and feelings of devaluation [21,80]. Offensive interpersonal behavior, such as incivility on the part of managers and colleagues or ageism at lower levels of intensity, such as harassment or a lack of respect due to age [81] may provoke reactions of stress, undermine older employees’ sense of occupational security, and damage their general functioning and psychological well-being.

And yet, we would like to suggest that cultivating a subjective perception of positive aging—which perceives aging as a natural process that includes self-acceptance of the disadvantages and advantages, the losses and the gains—may serve older employees by helping them to cope with age-related discrimination. Indeed, the research provides some support for this claim. For example, a study examining the relationship between perceptions of old age and subjective age found that those aged 50 and over, who held positive perceptions of old age, also demonstrated a lower chance of experiencing age discrimination [82]. In contrast, perceptions of age discrimination were found to be associated with a more negative perception of old age [83], an older subjective age [62], more depressive symptoms, lower levels of job satisfaction, and reduced subjective health [84]. Therefore, the third research hypothesis proposed is that in the second half of life, a relationship would be found between more positive perceptions of old age (i.e., a lower tendency to perceive aging as loss) and lower levels of perceived age discrimination in the workplace.

How do the three abovementioned psychological resources relate to one another and to discrimination at work? Studies have found that life satisfaction, which is one of the components of psychological well-being [85], was associated with a younger subjective age [86] and with more positive perceptions of aging. It was also related to taking preventive actions and improving health, such as exercising and avoiding smoking [87]. Similarly, a longitudinal study found that an older subjective age predicted less life satisfaction, when perceptions of aging were less positive, but not when perceptions of aging were more positive [88]. A similar finding was reported in an experimental setup that included two manipulations. In the first, participants felt older through a blurred visual stimulus; in the second, perceptions of aging were activated by reading sentences that included positive (“smart”) or negative (“weak”) age labels. Participants who felt older reported lower levels of life satisfaction when exposed to a stimulus evoking negative perceptions of old age, but not when positive perceptions of old age were activated in them [63]. This pattern indicates the significant effect of perceiving aging as loss on psychological well-being, compared to the more moderate effect of positive perceptions.

In light of the literature suggesting the existence of an independent contribution of each of these three mental resources to the perception of age discrimination at work, a uniform line is proposed for the interactive effect of these resources, which is based on a concept of compensation, rather than accumulation. According to the compensation approach, the existence of one resource compensates for the paucity of another resource and will therefore be more pronounced. This means that the resource’s contribution to a lower perception of age discrimination at work will be stronger when the additional resource or resources are weaker.

**Study design.** The study was designed to examine a wide age range of healthy, well-educated, and financially well Hebrew-speaking community-dwelling adults at the second half of life, most of whom who were still working. We aimed for a relatively equal gender distribution. A call for volunteering participation was published in several neighborhoods and workplaces by the research assistants, mostly at the center of the country. The questionnaires were online web-based questionnaires. The study model was a moderated moderation model, and the hypotheses were as follows:

**Hypotheses.** Three main effects will be discerned, that is, (H1) higher psychological well-being, (H2) younger subjective age, and (H3) lower perception of aging as a period of psychological losses, and each will be associated with lower workplace age discrimination. Two 2-way interactions were also hypothesized: (H4) Psychological well-being will moderate the association between subjective age and workplace age discrimination, so that among participants with lower psychological well-being, the older the subjective age, the higher the workplace age discrimination reported; (H5) Perception of aging as loss will moderate the association between subjective age and workplace age discrimination, so that among participants with higher perception of losses, the older the subjective age, the higher the reported workplace age discrimination. Finally, a 3-way interaction was hypothesized: (H6) Psychological well-being and the perception of aging as loss will moderate the association between subjective age and workplace age discrimination, so that among participants with lower psychological well-being and higher perceptions of losses, the older the subjective age, the higher the reported workplace age discrimination.

## 2. Materials and Methods

### 2.1. Sample

The study examined a convenience sample of 568 community-dwelling Israeli Jews. The mean age was 66.21 (SD = 11.95, range: 50–95), and 55.8% were women. The original sample included 582 participants, but 14 were excluded due to missing values. In terms of education, 12.9% had less than full high school education, 32.9% had full high school education, and 54.2% had higher education. In terms of marital status, 76.1% were married. Most of them reported good (48.4%) and very good health (21.0%), and very few not so good health (5.6%). A quarter (25.0%) defined their economic status as average, 5.6% as below the average, and 69.4% as above the average. Most of the participants were still working (67.1%).

### 2.2. Procedure

Data was collected from November 2019 to February 2020 (before the COVID-19 pandemic). Research assistants approached available participants in their neighborhoods and large workplaces and asked them if they were willing to voluntarily take part in the study. Inclusion criteria were being at the age of 50 or older, living in the community, having no formal cognitive impairment (this was assessed by asking them whether they were diagnosed with cognitive impairment or dementia) and speaking fluent Hebrew. Participants answered an online web-based questionnaire, mostly at their homes or at their workplace. Since the study was cross-sectional, attrition rate was negligible (14 participants were excluded due to missing values). Participants’ anonymity was kept since they were not asked to provide identifying details. They signed a digital informed consent before completing the questionnaires. The study received ethical approval by a departmental ethical review committee in the authors’ university (no. 0419).

### 2.3. Instruments

#### 2.3.1. Workplace Age Discrimination Scale

Workplace Age Discrimination Scale (WADS; [89]) measures workplace age discrimination. It consists of nine items (e.g., “I was given fewer opportunities to express my ideas because of my age”). Participants are asked to indicate how often they experienced each of the items on a scale ranging from 1 (*never*) to 5 (*very frequently*). The score is the average of ratings, and higher scores describe the respondent’s perception of being more often age-discriminated at work. The scale was validated for all age groups [90]. In the current sample, the scale demonstrated an excellent internal consistency (Cronbach’s alpha = 0.95).

#### 2.3.2. Psychological Well-Being

Psychological Well-Being was assessed using seven items taken from the Mental Health Continuum-Short Form [39], two items taken from Optimism–Pessimism scale [91] and a Single-Item Self-Esteem Scale [92]. The seven items included life satisfaction, self-acceptance, positive relationships, personal growth, autonomy, purpose in life, and mastery. For each item, participants rate how often they evidence each item on a scale ranging from 1 (*not at all*) to 5 (*all the time*). The items are then averaged, and higher scores reflect higher psychological well-being. The internal consistency coefficient was very good in the current sample (Cronbach’s alpha = 0.89).

#### 2.3.3. Subjective Age

Subjective age was measured based on Barak and Schiffman’s scale [93]. Participants are asked “Many people feel older or younger than their chronological age. How old do you feel?” in reference to four subjective age perceptions: mental age (“Mentally I feel as though I am”), physical age (“Physically, I feel as though I am”), appearance age (I look as though I am”), and behavior age (“I function as though I am”). Answers are given on a five-point scale, ranging from (1) “*much younger than my age*” to (5) “*much older than my age*”. The average score for the four items is calculated, and higher scores reflect an older subjective age. The internal consistency coefficient was very good (Cronbach’s alpha = 0.84).

#### 2.3.4. Aging as Loss

The attitudes to one’s aging as a period of psychological and social loss (e.g., “Old age is a depressing time of life”) were examined by four items from the short version of the Attitudes to Aging Questionnaire [94]. Participants were asked to rate the degree of their acceptance with each item, using a Likert scale ranging from 1 (*completely disagree*) to 5 (*completely agree*). Items were reversed (so that their agreement level reflected lower losses) and then averaged. The internal consistency coefficient was good in the current sample (Cronbach’s alpha = 0.81).

#### 2.3.5. Controlled Variables

Age, gender, education (rated on a scale ranging on a scale from 1 [*no formal education*] to 7 [*Master degree and above*]), marital status (categorized into 1=single, divorced or widow; 2 = married or cohabiting and recoded into 0 = non-married and 1 = married), employment (0 = unemployed, 1 = employed) and self-reported health (rated on a scale ranging on a scale from 1 [*not good at all*] to 5 [*very good*]) were included as covariates in all analyses.

### 2.4. Data Analysis

The statistical analyses were performed using SPSS 27. First, descriptive statistics and initial correlations of the study variables were computed. Then, in order to test the study hypotheses, multiple hierarchical regression analyses using SPSS 27 were conducted. Continuous predictors were mean-centered before analyses. Workplace age discrimination was regressed on covariates (age, gender, education, marital status, employment, and subjective health) in Step 1, on psychological well-being, subjective age, and on the losses scale of attitudes towards own aging in Step 2 (in order to examine main effects), on their respective three two-way interactions in Step 3, and on their three-way interaction in Step 4. Significant interactions were probed and plotted using the PROCESS 3.4 computational tool [24]. Potential multicollinearity between the predicting variables was rejected, as the tolerance and VIF ratios ranges were 0.65–0.98 and 1.03–1.54, respectively, which is in line with the literature requirements [95]. It should also be noted that the results of this study, i.e., all the significant effects, remained unchanged when the study model was re-examined without the covariates. A post hoc power analysis using G*power version 3.1.9.2 [96] for multiple regressions with 13 predictors, an effect size of 0.15, and n = 568 yielded a power of 1.00. Therefore, the current sample size was sufficient for discovering such effect sizes.

Finally, given the correlational nature of the study, additional analyses were conducted to investigate the possibility of inverse relationship between the dependent and independent variables, while acknowledging its limitation due to the cross-sectional nature of the study.

## 3. Results

Descriptive statistics and correlations for the study variables are presented in Table 1. In general, the mean level of workplace age discrimination was low, the mean level of subjective age tended to be younger than the actual age, and the mean level of psychological well-being in the current sample tended to be high. Several notable correlations can be seen in Table 1.

Table 2 presents the findings of the hierarchical regression analysis which examined our hypotheses. Workplace age discrimination was regressed on all variables.

In step 1, it was regressed on background variables (controlling for covariates). Gender, education, and self-reported health were negatively associated with workplace age discrimination (being a man, reporting higher education, and reporting better health were significantly associated with lower levels of workplace age discrimination). In step 2, it was also regressed on the three independent variables. While older subjective age only tended to be nearly positively associated with higher workplace age discrimination (Subjective age: B = 0.065, β = 0.070, *p* = 0.075), higher psychological well-being (Psychological well-being: B = −0.371, β = −0.371, *p* < 0.001) and the perception of aging as loss (B = −0.113, β = −0.124, *p* < 0.01) were negatively associated with the perception of workplace discrimination. Therefore, hypotheses 1 and 3 were confirmed, while hypothesis 2 was not supported by the findings. Out of the three respective two-way interactions (between psychological well-being and subjective age; between subjective age and aging a loss; between psychological well-being and aging a loss), only the psychological well-being and subjective age interaction was significant (B = −0.67, β = −0.107, *p* < 0.05).

**Figure 2:**
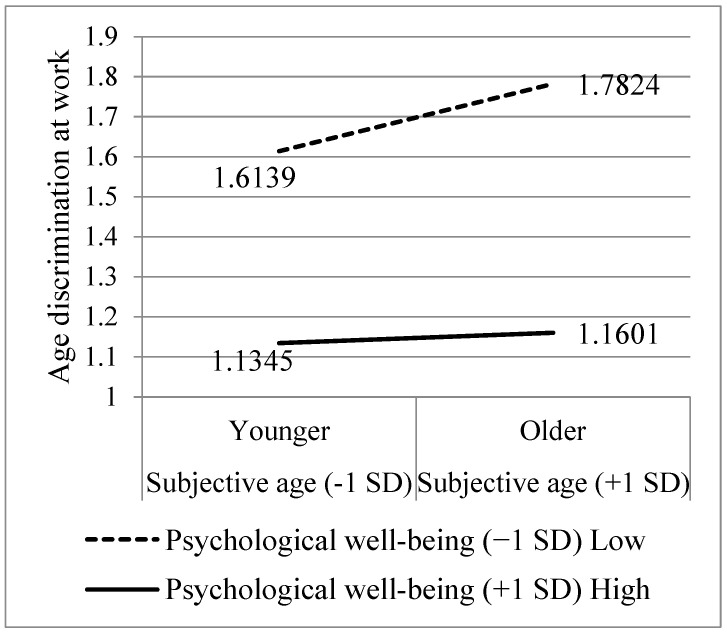
The Two-Way Interaction between Subjective Age and Psychological Well-being, Associated with Workplace Age Discrimination.

Figure 2 is presenting the results of probing this interaction. When probing this two-way interaction, it was demonstrated that while participants who reported lower psychological well-being (−1 SD) had a significant positive relationship between subjective age and workplace age discrimination (B = 0.169, *p* < 0.001; the steep black dashed curve), among participants who reported higher psychological well-being (+1 SD), the negative relationship was insignificant (B = 0.026, *p* = 0.5922; the black dashed line which is in parallel to the X axis). Moreover, from Figure 2, it can be seen that there is a clear main effect for psychological well-being (B = −0.376, *p* < 0.0001), showing that the reported level of age discrimination at work among participants who reported higher psychological well-being is low (no matter what their subjective age is), whereas the level of age discrimination at work among participants who reported lower psychological well-being is high, and grows higher, as their subjective age is older. This two-way interaction was qualified by a three-way interaction of subjective age, psychological well-being, and perception of aging as losses (B = −0.095, β = −0.190, *p* < 0.001).

**Figure 3:**
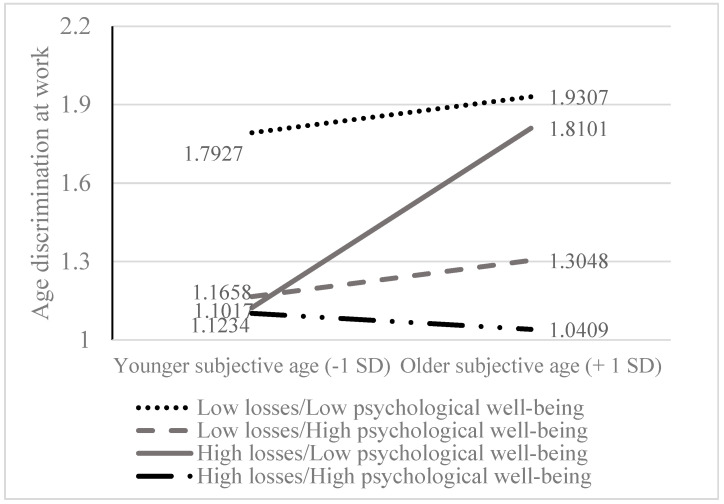
The Three-Way Interaction between Subjective Age, Psychological Well-being, and Aging as a loss Associated with Workplace Age Discrimination.

As depicted from Figure 3, only the steep black curve demonstrated significant positive association between subjective age and age discrimination at work (the older the subjective age, the higher the perception of age discrimination at work; B = −0.381, *p* < 0.0001). The steep black curve depicts participants reporting both higher psychological losses and lower psychological well-being, i.e., the most vulnerable participants. It can also be noted that while participants reporting lower psychological well-being and low perception of aging as loss (the curve with points) demonstrate the highest workplace age discrimination, no matter what their subjective age is, it is the combination of participants who are also reporting aging as loss which is responsible for the positive relations between subjective age and age discrimination at work. The other three curves (the curve with points, the dashed curve, and the curve with dashes and points) are almost parallel to the X axis and do not demonstrate significant associations between subjective age and age discrimination at work. The observation of these three curves and the probing of the 3-way interaction show that when psychological well-being is high (B = 0.068, *p* = 0.390) or the perception of aging as loss is low (B = 0.062, *p* = 0.262), or when psychological well-being is high and psychological losses is low (B = −0.042, *p* = 0.4359), in all these three cases, the relations between subjective age and age discrimination at work are insignificant.

In sum, while hypothesis 2 (subjective age would be positively associated with workplace age discrimination) and hypothesis 5 (the association between subjective age and workplace age discrimination would be moderated by the perception of aging as loss) were not supported, hypotheses 1 and 3 (higher psychological well-being, and lower perception of aging as loss will be associated with lower workplace age discrimination), hypothesis 4 (the association between subjective age and workplace age discrimination will be moderated by psychological well-being), and hypothesis 6 (the association between subjective age and workplace age discrimination wo;; be moderated by the combination of psychological well-being and the perception of aging as loss) were supported.

To determine if inverse relationships also exist between the study variables, additional hierarchical regression analysis was conducted. In this analysis, psychological well-being served as the dependent variable, while the covariates, which were added in step 1 to the regression, remained identical to those used in the previous analysis. The model did not show significance for the three-way interaction (R = 0.568, ∆R^2^ = 0.002, F (1, 525) = 1.194, *p* = 0.275). This analysis adds some empirical support for preferring age discrimination in the workplace as a dependent variable.

## 4. Discussion

The current study sought to examine the contribution of three psychological resources among adults in the second half of life, who are reporting age discrimination in the workplace. The research findings reveal that two resources (psychological well-being and the perception of aging as loss) contribute to the perceived age-related discrimination in the workplace, and that when examining the combined effects of these resources on perceptions of age-based discrimination at work, psychological well-being is the most important resource.

Regarding the main effects hypotheses, the negative relationship between and age discrimination at work (more psychological well-being, less age discrimination) was significant (H1), the positive relationship between subjective age and age discrimination at work had marginal significance (H2). The positive relationship between aging as loss and perceived age-based discrimination in the workplace were also significant (fewer losses, less perceived age-based discrimination, H3).

In line with previous studies, it was found that higher psychological well-being was associated with lower perceptions of age discrimination at work. A previous study also found that higher psychological well-being was associated with lower levels of age-based discrimination in the workplace, when there was also a sense of appreciation towards the older workers in the work environment [49]. Moreover, findings showed that employees with high psychological well-being at any age enjoy better mental and physical health [39,70] and function more effectively in the workplace [45]. Therefore, organizations need to encourage the cultivation of employees’ well-being in order to achieve higher productivity at work [46].

The importance of psychological well-being as a resource that moderates the perception of ageist discrimination in the workplace is particularly expressed in the confirmation of H4. The findings point to the possibility of interpreting the results through a compensation model. That is, availability of one resource (subjective age or psychological well-being) makes redundant the contribution of the other resource regarding the perception of age discrimination in the workplace. We hypothesized that among those who report lower psychological well-being, the younger the perceived subjective age, the less age discrimination at work will be reported. Conversely, among those with high reported psychological well-being, a low rate of age-based discrimination at work will be found—regardless of whether they felt younger or older than their age.

Next, we will offer two causal explanations for this finding. Nevertheless, it should be noted that these explanations require further research, as our cross-sectional design cannot support causality. First, psychological well-being is not life satisfaction or mere happiness, but is based on the eudaimonia philosophy devised by Aristotle, according to which the happy person is the good person, who brings forth that which is hidden within himself/herself [27]. Further to this conceptualization, it was found that older people (most of whom are retired) attribute the fact that they have worked throughout their lives as being related to higher levels of psychological well-being [50]. The workplace offers a rich context for personal development, enabling individuals to cultivate independence, acquire new skills, and expand their knowledge base. It also provides opportunities for self-reflection, leading to the identification of strengths and weaknesses while fostering a sense of purpose through daily work. In the second half of life, this development perhaps gives the employees—who can already envision the end of their professional career—a sense of value that protects them from the perception of age discrimination at work. It is also possible that such employees receive appreciation and recognition in terms of a self-fulfilling prophecy, and therefore also perceive themselves as suffering less from age discrimination at work.

Consistent with this line, studies have found that people with higher levels of psychological well-being are more efficient at work and their wages are higher, compared to those who report lower levels of psychological well-being (for a review, see [97]). In a related manner, higher meaning in work was found to be associated with general overall well-being [98]. Moreover, a positive link was found between generativity (i.e., where one’s concerns focus on others such as co-workers, peers, and community) and on higher satisfaction in life. This link was found to be mediated via increased meaning in work [99]. Therefore, it is likely that employees with high levels of psychological well-being are also valued and appreciated, and perceive themselves as having self-worth, self-confidence, ascribe meaning to their work, and suffer less from age discrimination at work.

It is possible that the weak support for hypothesis 2 indicates that younger subjective age is much more strongly related to perceptions of mental and physical health [66,68] than to perceived age discrimination in the workplace. In other words, young subjective age reflects more of an actual physical experience of vitality and health and may even stem more from biological variables, such as telomere length and gray matter density in the brain [100,101], than from an effort to defend against social perceptions that perceive older adults as being less valued than younger people. In this context, it may also be possible that the degree of stability in the perception of subjective age is responsible for the perception of age-based discrimination. Thus, when there is no stable perception of age, there is a greater tendency to attribute events at work as being related to age discrimination. Support for this interpretation can be found in a study which showed that fluctuations in subjective age indeed predicted more such negative age attributions among older workers aged 50–70 [73].

However, it is possible that while subjective age as a single variable only marginally contributes to perceptions of age discrimination at work, negative stereotypical perceptions of older age that are internalized and become negative self-perceptions of aging can contribute to an individual’s perception of an older subjective age. After being exposed to negative stereotypes from an early age, people adopt a negative labeling towards old age, and eventually direct it towards themselves [5]. In addition, they may attribute a young age to themselves starting from early adulthood [62]. Prior research investigated whether age discrimination affected subjective age. In that study, the relationship between these two variables was found to be indirect and mediated through negative self-perceptions of aging [102]. In our study, we did not measure mediation, and we cannot infer whether subjective age was indirectly affected by perceptions of age discrimination, and this should to be examined in future studies.

The findings of the present study also confirm H3, according to which lower perception of aging as loss was linked to lower levels of perceived age discrimination in the workplace. Accordingly, a study that examined employees aged 50 and over (37.7% employees) found that participants who perceived their aging in a more positive way reported less age discrimination [83]. The authors raised the possibility that participants with a more positive perception of aging are less likely to assume that any form of abusive behavior towards them is due to their age. The relationship between the two variables was also found in the complementary direction. A longitudinal study found that the experience of age discrimination at work predicted a decrease in positive self-perceptions of aging which, in turn, also led to an increase in depressive symptoms among older employees [83].

In addition, it can be suggested that here, too, a self-fulfilling prophecy is taking place. When older workers have positive perceptions about aging, and when they clearly do not attribute their difficulties at work to the notion that old age is a time of loss, their co-workers act accordingly and do not perceive the attitudes towards them in the context of their older age. This positive interaction maintains the older employees’ perceptions of aging as involving fewer losses. Therefore, they are not discriminated against on the basis of age in the workplace. This serves to reinforce employees’ perceptions of positive aging, and so forth. In addition to this explanation, it should be remembered that the current study’s data was collected only once (because of its cross-sectional study design). Thus, the study design does not allow us to draw conclusions about the causality described in this explanation, which can only be examined through a longitudinal study.

Our findings also support the importance of subjective perceptions of self-aging [6], as they demonstrate that subjective perceptions of self-aging are related to decreased perceptions of age discrimination in the workplace. These resources are anchored in the stream of positive psychology. This approach not only emphasizes a person’s psychological well-being as a central factor of the quality of his/her life [27] but also stresses the importance of the views of aging to better understand the ways in which aging individuals cope successfully with their social environment [103].

The main conclusion of the study is that individuals are more likely to perceive age discrimination in the workplace if their subjective age is old, if they perceive their old age as loss, and have low psychological well-being. Moreover, it is imperative to focus attention on vulnerable populations within society who may have such negative views. While the research findings show the importance of both psychological well-being and subjective perceptions of aging for the understanding of the phenomenon of perceived workplace age discrimination of older adults, the break-up of the moderated moderation model demonstrated, in accordance with H5 and H6, a compensation model. The model emphasized, first and foremost, the importance of high levels of psychological well-being as a kind of compensation for the lack of a younger subjective age and/or of lower perception of aging as loss. According to the compensation model, high psychological well-being is sufficient to allow an individual to hold a low perception of age discrimination at work, while low psychological well-being will manifest itself in a perception of age discrimination at work—even if the individual holds a perception of young subjective age, or of old age as involving less losses.

Despite controlling for subjective age, individuals with low perceptions of aging as loss and low psychological well-being reported high levels of perceived age discrimination (see the upper dotted line in Figure 3). While the construct of aging as loss, as measured by the perceived negative experiences associated with aging [94], is relevant to understanding age-related attitudes, its influence on workplace ageism may be less direct than that of psychological well-being. This construct primarily reflects general societal perceptions of aging and individuals’ passive compliance with these views, rather than perceptions that actively shape their workplace experiences. However, psychological well-being encompassed a broader range of workplace-relevant factors, such as mastery, personal growth, autonomy, and purpose [104]. Given the workplace’s central role in individuals’ lives, as a domain for responsibility, personal growth, and social contribution, it is plausible that psychological well-being, with its emphasis on these facets, is a more potent predictor of perceived age discrimination. Importantly, the triple interaction showed that the combination of low psychological well-being and the perception of aging as loss sharpens the contribution of older subjective age to the perception of age discrimination at work. Thus, among those individuals who lack the other two resources, subjective age becomes a central contributing factor for workplace age-based perceptions.

Therefore, we conclude that psychological well-being is an important defense against the perception of age discrimination in the workplace—its presence helps to minimize such perceptions, while its absence increases such perceptions. In the power relations between perceptions of aging and psychological well-being, it seems that in the context of age discrimination in the workplace, psychological well-being has the upper hand. In this context, future studies will be able to examine the mechanisms responsible for this connection and examine whether older employees, who enjoy a high level of psychological well-being, also maintain good employment relationships. The findings also demonstrate that when we examine the contribution of other perceptions of old age (subjective age and perceptions of aging) to perceived age discrimination at work, it is important to consider the individual’s psychological well-being.

The implication of these results can guide the creation of effective interventions to support older workers’ well-being and resilience against future workplace stressors, stemming from discriminatory behaviors by managers, co-workers, and clients [105]. Resilience can be achieved by helping employees view aging as a period of growth and opportunities rather than losses. Policymakers and organizations must take proactive steps to enact and implement anti-age discrimination legislation [84]. Moreover, organizations can develop innovative strategies for mitigating age-related workplace challenges, by providing their employees with opportunities for mindfulness training, which was proved affective among people over the age of 40 years, in improving their well-being through self-acceptance [106].

The present study has several limitations. First, although this study provides confirmation for a new model for self-age-based discrimination of older adults in the workplace, it should be regarded only as a preliminary study, as it is a cross-sectional investigation, which does not allow a causal directionality test between the tested variables. For example, it is impossible to be certain whether psychological well-being or subjective age are predictors or outcomes, because temporal precedence cannot be ascertained. Therefore, future research, using a cross-lagged longitudinal study design, is needed. Moreover, Zacher and Rudolph [107] found that the relationship between subjective age and positive work and life outcomes (e.g., task performance, career and life satisfaction) are confounded by core self-evaluations (i.e., how people feel about themselves). Although their research did not investigate age discrimination at the workplace, future research may consider testing the importance of self-core evaluation in this context. Additionally, the tested age range was very wide, and the sample also included people who are no longer working. Admittedly, although previous studies that examined similar issues were also based on even lower percentages of employees [82], and in spite of the fact that the age and the employment variables were controlled in our analyses, future studies are recommended to focus on narrower age ranges of older individuals who are still employed. In addition, the study was based on an Israeli sample; hence, there is a need for studies that reproduce the same results in other cultures.

Nevertheless, to the best of our knowledge, this study is the first to show the combined contribution of psychological well-being and two perceptions of aging (subjective age and the perception of old age as a period of losses) in relation to age discrimination in the workplace. This study also clarifies the great importance of psychological well-being of older employees in their career, as a resource that can greatly improve their perception of their last years at work and opens the door for many additional future studies on the subject.

## Figures and Tables

**Table 1:** Descriptive statistics and correlations between study variables.

	M	SD	1	2	3	4	5	6	7	8	9
1. Workplace age discrimination	1.44	0.74	-								
2. Psychological well-being	3.94	0.62	−0.45 ***	-							
3. Subjective age	2.28	0.80	0.23 ***	−0.32 ***	-						
4. Aging as loss	3.62	0.81	−0.33 ***	0.44 ***	−0.27 ***						
5. Age	66.21	11.95	0.10 *	0.01	0.04	0.02					
6. Gender (55.8% women) ^a^	-	-	−0.07	−0.06	0.08	−0.02 *	0.02				
7. Education	5.29	1.59	−0.26 ***	0.14 ***	−0.14 ***	0.16 ***	−0.04	−0.08			
8. Marital status (76.1% married) ^b^	-	-	−0.11 **	0.10 *	0.02	0.12 **	−0.08	−0.16 ***	0.11 **		
9. Employment (67.1% employed) ^c^	-	-	0.17 ***	−0.17 ***	0.11 **	−0.05	0.31***	0.08	−0.17 ***	−0.12 **	
10. Self-reported health	3.95	0.85	−0.35 ***	0.35 ***	−0.24 ***	0.25 ***	−0.14 ***	0.00	0.33 ***	0.16 ***	−0.30 ***

Note. N = 568. Correlation vlues represent Pearson coefficients except for coefficients for gender and marital status that represent point-biserial coefficients ad those for education that represent Spearman’s rank coefficients. ^a^ Coded 0 = man, 1 = woman, ^b^ Coded 0 = currently unmarrie, 1 = currently married. ^c^ Coded 0 = unemployed, 1 = employed.* *p* < 0.05, ** *p* ≤ 0.01, *** *p* < 0.001.

**Table 2:** Hierarchical Linear Regression Predicting Age Discrimination in the Workplace.

	B	β	*p*
Step 1: Covariates (∆R^2^ = 0.159)			
Age	0.002	0.029	0.497
Gender ^a^	−0.135	−0.091	0.026
Education	−0.079	−0.169	0.000
Marital status ^b^	−0.097	−0.056	0.170
Employment ^c^	0.07	0.04	0.307
Self-reported health	−0.223	−0.258	0.000
Step 2: Main effects (∆R^2^ = 0.139)			
Psychological Well-being	−0.371	−0.307	0.000
Subjective age	0.065	0.070	0.075
Aging as loss	−0.113	−0.124	0.003
Step 3: Two-way interactions (∆R^2^ = 0.009)			
Psychological Well-being X Subjective age	−0.067	−0.107	0.018
Psychological Well-being X Aging as loss	0.009	0.013	0.750
Subjective age X Aging as loss	0.034	0.048	0.263
Step 4: Three-way interaction (∆R^2^ = 0.021)			
Psychological Well-being X Subjective age X Aging as loss	−0.095	−0.190	0.000
*R^2^*= 0.312			

Note. N = 568. ^a^ Coded 0 = man, 1 = woman. ^b^ Coded 0 = currently unmarried, 1 = currently married. ^c^ Coded 0 = unemployed, 1 = employed.

## Data Availability

The data presented in this study are available upon request from the corresponding author.

## References

[1] United Nations Department of Economic and Social Affairs, Population Division (2022). World Population Prospects 2022: Summary of Results.

[2] Hertel G., Zacher H., Viswesvaran C., Anderson N., Ones D.S., Sinangil H.K. (2018). Managing an aging workforce. The SAGE Handbook of Industrial, Work, & Organizational Psychology.

[3] World Health Organization (2021). Global Report on Ageism: Executive Summary. https://www.who.int/publications/i/item/9789240020504.

[4] Ayalon L., Tesch-Römer C. (2018). Contemporary Perspectives on Ageism.

[5] Levy B. (2009). Stereotype embodiment: A psychosocial approach to aging. Curr. Dir. Psychol. Sci..

[6] Diehl M., Wahl H.W., Barrett A.E., Brothers A.F., Miche M., Montepare J.M., Westerhof G.J., Wurm S. (2014). Awareness of aging: Theoretical considerations on an emerging concept. Dev. Rev..

[7] Tabibian-Mizrachi Isor Haflaya Ba-Avoda al Reka Gil [Prohibition of Age Discrimination in the Workplace]. Knesset Research and Information Center, January 2007. https://main.knesset.gov.il/activity/info/research/pages/incident.aspx?ver=2&docid=d8bc8d55-f7f7-e411-80c8-00155d010977.

[8] Kim I.H., Noh S., Chun H. (2015). Mediating and moderating effects in ageism and depression among the Korean elderly: The roles of emotional reactions and coping responses. Osong Public Health Res. Perspect..

[9] Palmore E.B. (2004). Research note: Ageism in Canada and the United States. J. Cross. Cult. Gerontol..

[10] Chou R.J.A., Choi N.G. (2011). Prevalence and correlates of perceived workplace discrimination among older workers in the United States of America. Ageing Soc..

[11] Mourshed M., Jaffer A., Cashman H., Ruiz K.S., Sikes J. Meeting the World’s Midcareer Moment. Generation. https://www.generation.org/midcareer.

[12] Shah P., Kleiner B. (2005). New developments concerning age discrimination in the workplace. Equal Oppor. Int..

[13] Kite M.E., Stockdale G.D., Whitley B.E., Johnson B.T. (2005). Attitudes toward younger and older adults: An updated meta-analytic review. J. Soc. Issues.

[14] Henkens K. (2005). Stereotyping older workers and retirement: The managers’ point of view. Can. J. Aging/La Rev. Can. Du Vieil..

[15] Finkelstein L.M., Ryan K.M., King E.B. (2013). What do the young (old) people think of me? Content and accuracy of age-based metastereotypes. Eur. J. Work Organ. Psychol..

[16] Posthuma R.A., Campion M.A. (2007). Age stereotypes in the workplace: Common stereotypes, moderators, and future research Directions. J. Manag..

[17] Marcus J., Fritzsche B.A., Le H., Reeves M.D. (2016). Validation of the work-related age-based stereotypes (WAS) scale. J. Manag. Psychol..

[18] Palmore E., Palmore E.B., Branch L.G., Harris D.K. (2005). Costs of ageism. Encyclopedia of Ageism.

[19] Minnotte K.L. (2012). Perceived discrimination and work-to-life conflict among workers in the United States. Sociol. Quart..

[20] Orpen C. (1995). The effects of perceived age discrimination on employee job satisfaction, organizational commitment, and job involvement. Psychol. J. Hum. Behav..

[21] Redman T., Snape E. (2006). The consequences of perceived age discrimination amongst older police officers: Is social support a buffer?. Br. J. Manag..

[22] Broman C.L. (1997). Race-related factors and life satisfaction among African Americans. J. Black Psychol..

[23] Corning A.F. (2002). Self-esteem as a moderator between perceived discrimination and psychological distress among women. J. Couns. Psychol..

[24] Hayes A.F. (2018). Introduction to Mediation, Moderation, and Conditional Process Analysis: A Regression-Based Approach.

[25] World Health Organization (2001). The World Health Report 2001: Mental Health: New Understanding, New Hope.

[26] Ryff C.D. (1989). Happiness is everything, or is it? Explorations on the meaning of psychological well-being. J. Pers. Soc. Psychol..

[27] Ryff C.D. (2013). Psychological well-being revisited: Advances in the science and practice of eudaimonia. Psychother. Psychosom..

[28] Shmotkin D. (1990). Subjective well-being as a function of age and gender: A multivariate look for differentiated trends. Soc. Indic. Res..

[29] Springer K.W., Pudrovska T., Hauser R.M. (2011). Does psychological well-being change with age? Longitudinal tests of age variations and further exploration of the multidimensionality of Ryff’s model of psychological well-being. Soc. Sci. Res..

[30] Boyle P.A., Barnes L.L., Buchman A.S., Bennett D.A. (2009). Purpose in life is associated with mortality among community-dwelling older persons. Psychosom. Med..

[31] Czekierda K., Banik A., Park C.L., Luszczynska A. (2017). Meaning in life and physical health: Systematic review and meta-analysis. Health Psychol. Rev..

[32] Pinquart M. (2002). Creating and maintaining purpose in life in old age: A meta-analysis. Ageing Int..

[33] Kesavayuth D., Shangkhum P., Zikos V. (2022). Well-being and physical health: A mediation analysis. J. Happiness Stud..

[34] Ryff C.D., Radler B.T., Friedman E.M. (2015). Persistent psychological well-being predicts improved self-rated health over 9–10 years: Longitudinal evidence from MIDUS. Health Psychol. Open..

[35] Iwano S., Kambara K., Aoki S. (2022). Psychological interventions for well-being in healthy older adults: Systematic review and meta-analysis. J. Happiness Stud..

[36] van Dierendonck D., Lam H. (2023). Interventions to enhance eudaemonic psychological well-being: A meta-analytic review with Ryff’s Scales of Psychological Well-being. Appl. Psychol. Health Wellbeing.

[37] Neumeier L.M., Brook L., Ditchburn G., Sckopke P. (2017). Delivering your daily dose of well-being to the workplace: A randomized controlled trial of an online well-being programme for employees. Eur. J. Work Organ. Psychol..

[38] Keyes C.L., Dhingra S.S., Simoes E.J. (2010). Change in level of positive mental health as a predictor of future risk of mental illness. Am. J. Public Health.

[39] Lamers S.M., Westerhof G.J., Bohlmeijer E.T., ten Klooster P.M., Keyes C.L. (2011). Evaluating the psychometric properties of the mental health continuum-short form (MHC-SF). J. Clin. Psychol..

[40] Wood A.M., Joseph S. (2010). The absence of positive psychological (eudemonic) well-being as a risk factor for depression: A ten year cohort study. J. Affect. Disord..

[41] Ryff C.D., Singer B.H., Dienberg Love G. (2004). Positive health: Connecting well–being with biology. Philos. Trans. R. Soc. B Biol. Sci..

[42] Cohen R., Bavishi C., Rozanski A. (2016). Purpose in life and its relationship to all-cause mortality and cardiovascular events: A meta-analysis. Psychosom. Med..

[43] Ilmarinen J., Taylor P. (2013). Redesign of workplaces for an ageing society. Older Workers in an Ageing Society: Critical Topics in Research and Policy.

[44] Grawitch M.J., Gottschalk M., Munz D.C. (2006). The path to a healthy workplace: A critical review linking healthy workplace practices, employee well-being, and organizational improvements. Consult. Psychol. J. Pract. Res..

[45] Prochaska J.O., Evers K.E., Johnson J.L., Castle P.H., Prochaska J.M., Sears L.E., Rula E.Y., Pope J.E. (2011). The well-being assessment for productivity. J. Occup. Environ. Med..

[46] Schulte P.A., Guerin R.J., Schill A.L., Bhattacharya A., Cunningham T.R., Pandalai S.P., Eggerth D., Stephenson C.M. (2015). Considerations for incorporating “well-being” in public policy for workers and workplaces. Am. J. Public Health.

[47] Pascoe E.A., Smart Richman L. (2009). Perceived discrimination and health: A meta-analytic review. Psychol. Bull..

[48] Schmitt M.T., Branscombe N.R., Postmes T., Garcia A. (2014). The consequences of perceived discrimination for psychological well-being: A meta-analytic review. Psychol. Bull..

[49] Taylor P., McLoughlin C., Meyer D., Brooke E. (2013). Everyday discrimination in the workplace, job satisfaction and psychological wellbeing: Age differences and moderating variables. Ageing Soc..

[50] Ryff C.D., Heidrich S.M. (1997). Experience and well-being: Explorations on domains of life and how they matter. Int. J. Behav. Dev..

[51] Sanders C.A., Rose H., Booker J.A., King L.A. (2021). Claiming the role of positive psychology in the fight against prejudice. J. Posit. Psychol..

[52] Kushlev K., Drummond D.M., Heintzelman S.J., Diener E. (2019). Do happy people care about society’s problems?. J. Posit. Psychol..

[53] Ejova A., Krátký J., Kundtová Klocová E., Kundt R., Cigán J., Kotherová S., Bulbulia J., Gray R.D. (2021). The awe-prosociality relationship: Evidence for the role of context. Relig. Brain Behav..

[54] Womick J., Ward S.J., Heintzelman S.J., Woody B., King L.A. (2019). The existential function of right-wing authoritarianism. J. Pers..

[55] Albaghli B., Carlucci L. (2020). The link between Muslim religiosity and negative attitudes toward the west: An Arab study. Int. J. Psychol. Relig..

[56] Cantal C., Milfont T.L., Wilson M.S., Gouveia V.V. (2015). Differential effects of right–wing authoritarianism and social dominance orientation on dimensions of generalized prejudice in Brazil. Eur. J. Pers..

[57] Hodson G., MacInnis C.C., Busseri M.A. (2017). Bowing and kicking: Rediscovering the fundamental link between generalized authoritarianism and generalized prejudice. Pers. Individ. Differ..

[58] Naegele L., De Tavernier W., Hess M., Ayalon L., Tesch-Römer C. (2018). Work environment and the origin of ageism. Contemporary Perspectives on Ageism.

[59] Kornadt A.E., Weiss D., De Paula Couto M.C., Rothermund K. (2023). Internalization or dissociation? Negative age stereotypes make you feel younger now but make you feel older later. J. Gerontol. B Psychol. Sci. Soc. Sci..

[60] Westerhof G.J., Miche M., Brothers A.F., Barrett A.E., Diehl M., Montepare J.M., Wahl H., Wurm S. (2014). The influence of subjective aging on health and longevity: A meta-analysis of longitudinal data. Psychol. Aging.

[61] Pinquart M., Wahl H.-W. (2021). Subjective age from childhood to advanced old age: A meta-analysis. Psychol. Aging.

[62] Stephan Y., Sutin A.R., Terracciano A. (2015). How old do you feel? The role of age discrimination and biological aging in subjective age. PLoS ONE.

[63] Eibach R.P., Mock S.E., Courtney E.A. (2010). Having a “senior moment”: Induced aging phenomenology, subjective age, and susceptibility to ageist stereotypes. J. Exp. Soc. Psychol..

[64] Barrett A.E., Gumber C. (2018). Feeling old, body and soul: The effect of aging body reminders on age identity. J. Gerontol. B Psychol. Sci. Soc. Sci..

[65] Bellingtier J.A., Neupert S.D., Kotter-Grühn D. (2015). The combined effects of daily stressors and major life events on daily subjective ages. J. Gerontol. B Psychol. Sci. Soc. Sci..

[66] Kotter-Grühn D., Neupert S.D., Stephan Y. (2015). Feeling old today? Daily health, stressors, and affect explain day-to-day variability in subjective age. Psycholo. Health.

[67] Weiss D., Weiss M., Zacher H., Rudolph C.W. (2022). Beyond chronological age: Alternative age constructs and their implications in the work context. Age and Work: Advances in Theory, Methods, and Practice.

[68] Kotter-Grühn D., Kornadt A.E., Stephan Y. (2016). Looking beyond chronological age: Current knowledge and future directions in the study of subjective age. Gerontology.

[69] Stephan Y., Caudroit J., Chalabaev A. (2011). Subjective health and memory self-efficacy as mediators in the relation between subjective age and life satisfaction among older adults. Aging Ment. Health.

[70] Keyes C.L., Westerhof G.J. (2012). Chronological and subjective age differences in flourishing mental health and major depressive episode. Aging Ment. Health.

[71] Rudolph C.W., Kunze F., Zacher H. (2019). Getting objective about subjective age: Introduction to a special issue. Work. Aging Retire..

[72] Shane J., Hamm J.M., Heckhausen J. (2019). Subjective age at work: Feeling younger or older than one’s actual age predicts perceived control and motivation at work. Work Aging Retire..

[73] Armenta B.M., Scheibe S., Stroebe K., Postmes T., Van Yperen N.W. (2018). Dynamic, not stable: Daily variations in subjective age bias and age group identification predict daily well-being in older workers. Psychol. Aging.

[74] Baltes M.M., Lang F.R. (1997). Everyday functioning and successful aging: The impact of resources. Psychol. Aging.

[75] Carstensen L.L., Isaacowitz D.M., Charles S.T. (1999). Taking time seriously: A theory of socioemotional selectivity. Am. Psychol..

[76] Charles S.T. (2010). Strength and vulnerability integration: A model of emotional well-being across adulthood. Psychol. Bull..

[77] Reed A.E., Chan L., Mikels J.A. (2014). Meta-analysis of the age-related positivity effect: Age differences in preferences for positive over negative information. Psychol. Aging.

[78] Scheibe S., Carstensen L.L. (2010). Emotional aging: Recent findings and future trends. J. Gerontol. B Psychol. Sci. Soc. Sci..

[79] Ashkanasy N.M., Dorris A.D. (2017). Emotions in the workplace. Annu. Rev. Organ. Psychol. Organ. Behav..

[80] Colella A.J., McKay P.F., Daniels S.R., Signal S.M., Kozlowski S.W.J. (2012). Employment discrimination. The Oxford Handbook of Organizational Psychology.

[81] Cortina L.M., Magley V.J., Williams J.H., Langhout R.D. (2001). Incivility in the workplace: Incidence and impact. J. Occup. Health Psychol..

[82] Giasson H.L., Queen T.L., Larkina M., Smith J. (2017). Age group differences in perceived age discrimination: Associations with self-perceptions of aging. Gerontologist.

[83] Han J., Richardson V.E. (2015). The relationships among perceived discrimination, self-perceptions of aging, and depressive symptoms: A longitudinal examination of age discrimination. Aging Ment. Health.

[84] Marchiondo L.A., Gonzales E., Williams L.J. (2019). Trajectories of perceived workplace age discrimination and long-term associations with mental, self-rated, and occupational health. J. Gerontol. B Psychol. Sci. Soc. Sci..

[85] Diener E., Suh E.M., Kahneman D., Diener E., Schwarz N. (1999). National differences in subjective wellbeing. Well-Being: The Foundations of Hedonic Psychology.

[86] Zacher H., Palgi Y., Shrira A., Diehl M. (2022). Subjective views of aging at work and in the retirement transition. Subjective Views of Aging: Theory, Research, and Practice.

[87] Bryant C., Bei B., Gilson K., Komiti A., Jackson H., Judd F. (2012). The relationship between attitudes to aging and physical and mental health in older adults. Int. Psychogeriatr..

[88] Mock S.E., Eibach R.P. (2011). Aging attitudes moderate the effect of subjective age on psychological well-being: Evidence from a 10-year longitudinal study. Psychol. Aging.

[89] Marchiondo L.A., Gonzales E., Ran S. (2016). Development and validation of the workplace age discrimination scale. J. Bus. Psychol..

[90] Lagacé M., Firzly N., Zhang A., Luszczynska M. (2020). Self-report measures of ageism in the workplace. Researching Ageing.

[91] Kemper C.J., Beierlein C., Kovaleva A., Rammstedt B. (2013). Development and validation of an ultra-short measure for the construct of optimism-pessimism-The Scale Optimism-Pessimism-2 (SOP2). Diagnostica.

[92] Robins R.W., Hendin H.M., Trzesniewski K.H. (2001). Measuring global self-esteem: Construct validation of a single-item measure and the Rosenberg Self-Esteem Scale. Pers. Soc. Psychol. Bull..

[93] Barak B., Schiffman L.G. (1981). Cognitive age: A Nonchronological age variable. Adv. Consum. Res..

[94] Laidlaw K., Kishita N., Shenkin S.D., Power M.J. (2018). Development of a short form of the Attitudes to Ageing Questionnaire (AAQ). Int. J. Geriatr. Psychiatry.

[95] O’brien R.M. (2007). A caution regarding rules of thumb for variance inflation factors. Qual. Quant..

[96] Faul F., Erdfelder E., Buchner A., Lang A. (2009). Statistical power analyses using G*Power 3.1: Tests for correlation and regression analyses. Behav. Res. Methods.

[97] Lyubomirsky S., King L., Diener E. (2005). The benefits of frequent positive affect: Does happiness lead to success?. Psychol. Bull..

[98] Arnold K.A., Turner N., Barling J., Kelloway E.K., McKee M.C. (2007). Transformational leadership and psychological well-being: The mediating role of meaningful work. J. Occup. Health Psychol..

[99] Shilo-Levin S., Shrira A., Hoffman Y. (2021). Feeling older can be advantageous: A study on Generativity, meaning in work and life satisfaction in Israeli workplaces. J. Happiness Stud..

[100] Chen W., Kimura M., Kim S., Cao X., Srinivasan S.R., Berenson G.S., Kark J.D., Aviv A. (2011). Longitudinal versus cross-sectional evaluations of leukocyte telomere length dynamics: Age-dependent telomere shortening is the rule. J. Gerontol. Ser. A Biomed. Sci. Med. Sci..

[101] Kwak S., Kim H., Chey J., Youm Y. (2018). Feeling how old I am: Subjective age is associated with estimated brain age. Front. Aging Neurosci..

[102] Marquet M., Chasteen A.L., Plaks J.E., Balasubramaniam L. (2019). Understanding the mechanisms underlying the effects of negative age stereotypes and perceived age discrimination on older adults’ Well-being. Aging Ment. Health.

[103] Palgi Y., Shrira A., Diehl M. (2022). Subjective Views of Aging: Theory, Research, and Practice.

[104] Steger M., Yeoman R., Bailey C., Madden A., Thompson‏et M. (2019). Meaning in Life and in Work. The Oxford Handbook of Meaningful Work.

[105] Ford M.T., Matthews R.A., Wooldridge J.D., Mishra V., Kakar U.M., Strahan S.R. (2014). How do occupational stressor-strain effects vary with time? A review and meta-analysis of the relevance of time lags in longitudinal studies. Work Stress.

[106] Mahlo L., Windsor T.D. (2020). Older and more mindful? Age differences in mindfulness components and well-being. Aging Ment. Health.

[107] Zacher H., Rudolph C.W. (2018). Just a mirage: On the incremental predictive validity of subjective age. Work Aging Retire..

